# Risk factors of hepatic function alterations in hospitalized adult patients treated with short-term parenteral nutrition receiving the same lipid composition at the same dose

**DOI:** 10.1186/s12944-018-0912-4

**Published:** 2018-11-24

**Authors:** Josep Llop-Talaveron, Maria B. Badia-Tahull, Toni Lozano-Andreu, Ana Suarez-Lledo, Elisabet Leiva-Badosa

**Affiliations:** Pharmacy Department, Hospital Universitari Bellvitge, IDIBELL, Universitat Barcelona, c/Feixa Llarga s/n 08917 L’Hospitalet de Llobregat, Barcelona, Spain

**Keywords:** Liver tests, Parenteral nutrition, Lipid emulsion, Non-critical patient, Risk factor

## Abstract

**Background:**

High doses and vegetable origin of lipid emulsions (LE) are prominent factors for liver test (LT) alterations in patients treated with parenteral nutrition (PN). This study aims to determine incidence of LT alterations, and risk factors related to these alterations in patients with short term PN with homogenous LE.

**Methods:**

Adult non-critically ill hospitalized patients, with normal LTs at the beginning of PN, receiving 0.8 g/kg/day of an olive/soybean LE were included. A paired Student t-test was applied to compare final with initial LT values. LT variation (end vs start of PN) according to type of surgery and infection was studied by means of an analysis of the variance. Univariate and multivariate analyses were constructed to relate the variations of each of the 4 LTs with the adjustment variables.

**Results:**

One hundred eighty one patients (66.57 ± 12.89 years; 72.4% men), 66.8% suffered from cancer. Final LT values increased from initial values for gamma-glutamyltransferase (GGT) 2.69 ± 2.49 μkat/L vs 0.55 ± 0.36 μkat/L, alkaline phosphatase (AP) 1.97 ± 1.49 μkat/L vs 1.04 ± 0.33 μkat/L, and alanine aminotransferase (ALT) 0.57 ± 0.92 μkat/L vs 0.32 ± 0.26 μkat/L. GGT and AP variations were associated with days of PN; GGT, AP and total bilirubin with surgical patients, AP variations with infection, and GGT with cancer. Multivariate analysis: elevation of GGT, AP and ALT was related to infection, days of PN and surgery.

**Conclusions:**

Factors that increased the risk of LTs elevation during short term PN treatment were duration of PN, surgery, cancer, and infection associated with oxidative stress.

## Introduction

Alterations of liver tests (LTs), common in patients with parenteral nutrition (PN), have a multifactorial component. There are associated factors, such as the basal clinical state (liver or biliary disease, abdominal surgery and sepsis) and pharmacological treatments (hepatotoxic drugs, transfusions), but there are also factors inherent in PN itself and in lack of oral intake [1–3]. In short-term PN, these alterations usually revert upon discontinuation of nutritional therapy and are not associated with liver damage. Clinical manifestations, such as cholestasis or steatosis, have been described mainly after prolonged periods of PN especially in premature infants.

An association between the administration of lipid emulsions (LE) and the alterations of LTs has been described. Some studies related this alteration to high doses of LE [4, 5] and others to the emulsions’ vegetable origin [6–8]. Our group has studied the impact of vegetal LE on the alteration of LTs, as well as the protective effect of fish oil, and our findings support the hypothesis of an association between liver damage and use of vegetal LE [9, 10] due, in part, to plasma phytosterol concentration. This association is relatively well established in premature infants and adults with long-term PN, as well as those on home PN (HPN) [11]. Adult inpatients often receive PN during the acute phase of their disease, PN thereby coexisting with clinical situations that also alter LTs. The diversity of clinical situations and the different types of nutrient used make it difficult to differentiate and establish the weight and importance of potentially hepatotoxic factors. The recognition of risk factors associated with LT alterations, as well as early detection of impaired hepatic function with specific indicators, may allow better nutritional design for prevention and/or treatment of these alterations.

The objective of this study was to determine in non-critically ill adult inpatients treated with short term PN with an olive/soybean LE at a fixed dose of 0.8 g/kg/day, without oral/enteral support:

- The incidence of LT alterations [gamma-glutamyl transferase (GGT), alkaline phosphatase (AP), alanine aminotransferase (ALT) and total bilirubin (TB)].
- Demographic and clinical factors, independent of LE type, related to the LT alterations.

## Methods

### Population and follow-up time

The study included adult hospitalized patients with normal LTs at the beginning of PN who received a lipid intake of 0.8 g/kg/day of an olive/soybean LE (Clinoleic®). Patients without total biochemical and hematological values at baseline, those with plasma triglycerides ≥3 mmol/L, pregnant women, nursing mothers, transplanted patients, patients admitted to critical units, and patients receiving corticosteroids or immuno-suppressants in the month prior to the study were excluded.

Patients were studied during the days that they maintained a lipid intake of 0.8 g/kg/day with olive oil/soybean (Clinoleic®) without supplemental oral or enteral nutrition. Follow-up was discontinued if there was a change of lipid dose for any reason, including increased triglyceridemia (≥3 mmol/L), and transfer to critical units.

### Variables

Demographic and clinical variables were collected: age, height, weight and diagnosis.

As dependent variables, LTs were studied taking as reference the following normal values (calculated as the average of the reference values of our hospital): GGT < 1.11 μkat/L; AP < 1.5 μkat/L; ALT < 0.73 μkat/L; TB < 17 mmol/L.

The following adjustment variables were used:

- Demographic: sex, body mass index (BMI)
- Clinical: diagnostic, infection, surgery and cancer
- Plasmatic safety and follow-up parameters: creatinine, triglycerides, glucose, albumin, C- reactive protein (CRP), leukocytes, lymphocytes, platelet count and prothrombin time
- Nutritional data: days of PN

### Statistics

To compare final with initial LT values, a paired Student t-test was applied.

LT variations (differences between the values at the start and the end of the study) according to the type of surgery and infection localization were studied by means of an analysis of variance (ANOVA).

Univariate analyses were constructed to compare the variations of each of the four LTs (differences between the values at the end of the study and the initial values) with each one of the adjustment variables:

- Linear regression was used for the continuous variables (days of study, CRP, BMI, triglycerides, glycemia, leukocytes, lymphocytes, creatinine, albumin, platelets and prothrombin time)
- Student t-test for binary categorical variables (sex and cancer)
- ANOVA for each of the three-categorical variables of infection and surgery classified as not present, present at the beginning of PN or developed during PN treatment.

A multivariate approach was developed to study the relationship between LT variations and the risk factors studied; a stepwise linear regression model with an inclusion criterion of *p* < 0.1 was used for all variables.

## Results

A total of 181 patients with the diagnoses shown in Table 1 were included in the study; 66.8% of patients had neoplasms. The most prevalent diagnosis, representing 60.2% of the cases, was digestive cancer: colonic (18.2%), rectum (17.1%), gastric (13.3%) and esophagus (6.1%).

**Table 1:** Diagnostics

Diagnostic	*n*	%
Digestive cancer	109	60.2
Non digestive cancer	12	6.6
Fistula	9	5.0
Diverticulitis	9	5.0
Abdominal adhesions	8	4.4
Digestive occlusion	6	3.3
Inflammatory bowel disease	6	3.3
Others	22	12.2
Total	181	100

Table 2 shows demographic, clinical and nutritional characteristics together with analytical values at the beginning of PN and at the end of follow-up. Overall, 79.6% of patients were surgical cases and 37% had infections. Among plasmatic parameters studied, except for albumin and leukocytes, statistically significant differences were found between the initial and final values of the study. Except for GGT and AP, final values of the analytical variables were within normal range.

**Table 2:** Demographic, clinical, nutritional and analytical characteristics

Demographics	Value		
Men (*n*, %)	131 (72.4)		
Age (years±SD)	66.57 ± 12.89		
Weight (Kg ± SD)	68.79 ± 13.15		
Height (cms ± SD)	166.00 ± 8.85		
Clinical data			
Cancer (patients: *n*, %)	121 (66.8)		
Infection (patients: *n*, %)	67 (37.0)		
Infection before PN treatment (focus: *n*, %)	35 (19.3)		
Infection during PN treatment (focus: *n*, %)	50 (27.6)		
Type of infection:			
Abdominal (patients: *n*, %)	26 (30.9)		
Surgical wound (patients: *n*, %)	17 (20.2)		
Urinary (patients: *n*, %)	18 (21.4)		
Pulmonary (patients: *n*, %)	9 (10.7)		
Bacteriemia (patients: *n*, %)	14 (16.7)		
Surgery (patients: *n*, %)	144 (79.6)		
Surgery before PN treatment (patients: *n*, %)	139 (76.8)		
Surgery during PN treatment (patients: *n*, %)	12 (6.6)		
Nutritional data			
Days of follow-up (days ±SD)	8.43 ± 6.64		
Analytical variables	Beginning of PN Average ± SD	End of follow-up Average ± SD	t-pairs *p*
Gamma-glutamyl transferase (μkat/L)	0.55 ± 0.36	2.69 ± 2.49	0.000
Alkaline phosphatase (μkat/L)	1.04 ± 0,33	1.97 ± 1.49	0.000
Alanine aminotransferase (μkat/L)	0.32 ± 0.26	0.57 ± 0.92	0.000
Total bilirubin (μmol/L)	6.98 ± 4.55	6.08 ± 5.37	0.029
C-reactive protein (mg/L)	139.23 ± 111.99	99.72 ± 108.81	0.000
Albumin (g/L)	28.44 ± 5.50	29.14 ± 5.55	0.061
Leucocytes (x10E9/L)	10.79 ± 5.27	10.45 ± 4.89	0.427
Lymphocytes (x10E9/L)	1.02 ± 0.52	1.31 ± 1.21	0.000
Triglycerides (mmol/L)	1.42 ± 0.66	1.85 ± 0,72	0.000
Glucose (mmol/L)	7.02 ± 2.44	6.44 ± 1.81	0.000
Creatinine (μmol/L)	78.94 ± 53.61	71.27 ± 46.69	0.000
Platelets (x10E9/L)	270.01 ± 108.14	370.98 ± 159.14	0.000
Prothrombin time	1.18 ± 0,15	1.16 ± 0,13	0.014

*SD* standard deviation

**Fig. 1:**
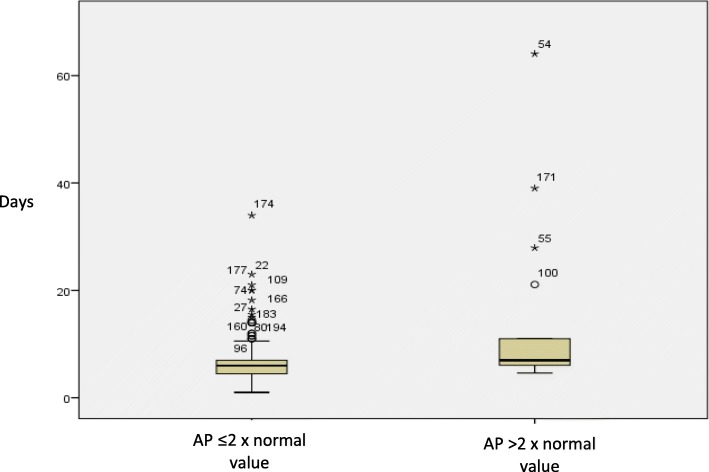
Days in treatment with parenteral nutrition and alkaline phosphatase values. Mean ± standard deviation of days of parenteral nutrition in patients that doubled the normal value of alkaline phosphatase vs those that did not: 13.94 ± 15.03 vs 6.81 ± 3.75

**Fig. 2:**
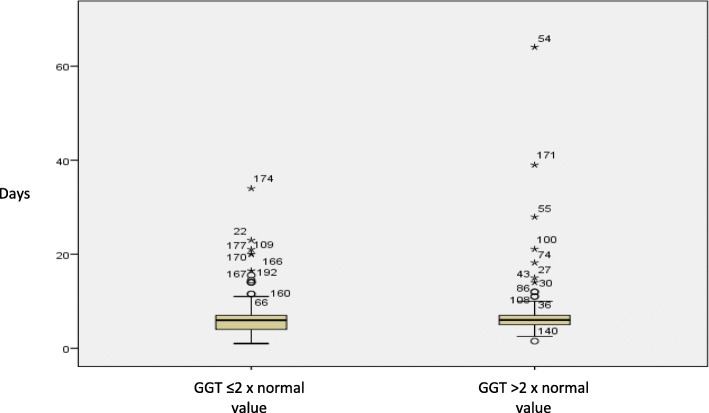
Days in treatment with parenteral nutrition and Gamma Glutamyl Transferase values. Mean ± standard deviation of days of parenteral nutrition in patients that doubled the normal value of gamma glutamyl transferase vs those that did not: 7.89 ± 8.03 vs 7.03 ± 5.2 *p* > 0.05

Elevation of LT final values was studied, taking into account 3 scenarios: elevation compared to the initial values; final values above normal limits; and final values doubling initial values (Table 3). GGT and AP were the most frequently elevated LTs. GGT elevations appeared earlier than AP, which tended to occur more gradually over the course of PN (Figs. 1 and 2).

**Table 3:** Liver function parameter elevations

	Patients with final values higher than initial values		Patients with final values higher than normal values		Patients with final values higher than 2-fold the initial values	
	*N*	%	*n*	%	*n*	%
GGT	175	96.7	131	72.4	85	47.0
AP	170	93.9	107	59.1	19	10.5
ALT	122	67.4	33	18.2	7	3.9
TB	62	34.3	3	1.7	2	1.1

*GGT* Gamma-glutamyl transferase (μkat/L)*, AP* Alkaline phosphatase (μkat/L)*, ALT* Alanine aminotransferase (μkat/L), *TB* Total bilirrubin (μmol/L)

Sixty-seven patients had infections confirmed by a positive culture (37%). Eighty-four infection focuses were detected because 18 patients (9.9%) presented with more than one infection focus. No statistically significant differences were found studying LT variations in relation to the origin of the infection (data not shown). Meanwhile statistically significant differences were found in the relationship between different types of surgery and the variation of AP, whereas for GGT there was only a tendency to signification (Table 4). The major AP and GGT increases were found for small bowel resection and gastroesophageal surgery, respectively.

**Table 4:** ANOVA showing the variation of liver parameters GGT and AP and types of surgery: differences between the end and beginning of the study

Surgery		GGT variations	AP variations
	*n*	Average ± SD	Average ± SD
No surgery	41	1.25 ± 2.02	0.57 ± 1.03
Colorectal	77	2.35 ± 2.58	0.87 ± 0.93
Gastroesophagic	30	2.91 ± 2.67	1.07 ± 0.81
Bilio-hepatic and pancreatic	7	1.35 ± 2.41	0.46 ± 0.29
Small intestine and peritoneal	17	2.41 ± 2.57	2.07 ± 3.80
Others [not digestive system]^a^	9	1.99 ± 2.18	0.91 ± 0.83
Total	181	2.14 ± 2.45	0.94 ± 1.48
*F Snedecor (sig.)*		*2.04 (0.076)*	*2.84 (0.017)*

*GGT* Gamma-glutamyl transferase (μkat/L), *AP* Alkaline phosphatase (μkat/L), *SD* standard deviation, *ANOVA* analysis of variance

^a^Others [not digestive system]: radical hysterectomy, cystoprostatectomia, resection of bladder, lymphadenectomy, prosthesis implant, nephrectomy

Table 5 shows the univariate study of LT variations and each of the variables studied:

- Among the clinical variables, surgery showed statistically significant differences in GGT, AP and TB variations; infection and cancer were significantly associated with variations of AP with GGT, respectively.
- CRP was the only plasmatic parameter that showed statistically significant differences being associated only with AP variations.
- For nutritional parameters, the number of days of PN was associated with GGT and AP variations.
- Leukocytes at the end of study were associated with BIL variations.
- Body Mass Index, Triglycerides, Glycemia, Lymphocytes, Creatinine, Albumin, Platelets Prothrombin time and sex did not show statistically significant differences in any LT.

**Table 5:** Univariate analysis showing liver test variations and risk factors: differences between the beginning and end of treatment

LT variations	R^2^	B	95% CI	p Linear regression
Days of follow-up (with PN)				
GGT	0.041	0.074	1.045–2.117	0.006
AP	0.372	0.135	0.109–0.161	0.000
ALT	0.001	0.004	−0.015-0.024	0.807
TB	0.018	0.101	−0.010-0.213	0.075
CRP at the end of study				
GGT	0.009	0.002	−0.001–0.005	0.217
AP	0.043	0.003	0.001–0.005	0.005
ALT	0.000	0.000	−0.001-0.001	0.847
TB	0.002	0.002	−0.009-0.005	0.581
Leukocytes at the end of study				
GGT	0.011	0.053	−0.020-0.126	0.157
AP	0.011	0.611	−0.013-0.075	0.168
ALT	0.006	0.014	−0.013-0.040	0.313
TB	0.026	0.166	0.014–0.318	0.032
Cancer				
LT variations	No (*n* = 60) Mean ± SD		Yes (*n* = 121) Mean ± SD	p student t-test
GGT	1.48 ± 1.49		2.48 ± 2.76	0.002
AP	0.77 ± 0.97		1.02 ± 1.68	0.283
ALT	0.38 ± 1.42		0.18 ± 0.41	0.293
TB	−0.89 ± 6.69		6.69 ± 4.14	0.815
Infection				
LT variations	No (*n* = 114) Mean ± SD	Beginning PN (*n* = 17) Mean ± SD	During PN (*n* = 50) Mean ± SD	p ANOVA
GGT	1.96 ± 2.67	1.76 ± 1.39	2.69 ± 2.14	0.173
AP	0.76 ± 0.93^b^	0.71 ± 0.62	1.42 ± 2.36^b^	0.025
ALT	0.16 ± 0.40	0.31 ± 0.68	0.42 ± 1.52	0.223
TB	−1.54 ± 4.18	−0.58 ± 3.15	0.18 ± 7.06	0.184
Surgery				
LT variations	No (*n* = 37) Mean ± SD	Beginning PN (*n* = 132) Mean ± SD	During PN (*n* = 12) Mean ± SD	P ANOVA
GGT	1.01 ± 1.54^a,b^	2.38 ± 2.56^a^	3.06 ± 2.60^b^	0.004
AP	0.48 ± 0.99^b^	0.94 ± 0.99^c^	2.34 ± 4.27^b,c^	0.001
ALT	0.25 ± 0.44^b^	0.25 ± 1.00	0.22 ± 0.48	0.993
TB	−2.26 ± 4.98^b^	−0.95 ± 5.19	1.93 ± 3.06^b^	0.044

*GGT* Gamma-glutamyl transferase (μkat/L), *AP* Alkaline phosphatas (μkat/L) e, *ALT* Alanine aminotransferase (μkat/L), *TB* Total bilirubin (μmol/L), *SD* standard deviation, *CRP* C-reactive protein, *PN* parenteral nutrition. R2 determination coefficient B: constant in the statistical model ^a^: differences between no condition and condition at the beginning of PN, ^b^: differences between no condition and condition during PN treatment, ^c^: statistically significant differences between the condition at the beginning of PN and the condition appearing during PN treatment

After obtaining these results we chose a stepwise multiple linear regression model with an inclusion criterion of *p* < 0.1 for all variables in order to achieve maximum explanatory models.

The results of the multivariate analysis are shown in Table 6. Variation of AP presents the highest determination coefficient (R^2^ 0.523) and includes 5 variables. GGT variation presents a R^2^ 0.150 and includes 4 variables. The TB and ALT variations have 0.089 and 0.05 R^2^ values and include 3 and 1 variable, respectively.

**Table 6:** Multiple lineal regressions: differences between liver parameter variations and risk factors (*p* < 0.1)

Variable	GGT variation		AP variation		ALT variation		Bilirubin total variation	
	B [95% CI]	*p*	B [95% CI]	*p*	B [95% CI]	*p*	B [95% CI]	*p*
Surgery	**1.05 [0.29–1.80]**	**0.007**	**0.18 [0.14–0.84]**	**0.006**			**2.00 [0.14–0.40]**	**0.014**
Days in study	**0.08 [0.03–0.13]**	**0.003**	**0.12 [0.18–0.17]**	**0.000**			0.10 [−0.01–0.21]	0.072
Cancer	**1.06 [0.27–1.84]**	**0.009**	0.178 [−0.15–0.69]	0.060				
Infection	0.44 [−0.01–0.88]	0.056	**0.162 [0.04–0.68]**	**0.027**	**0.08 [0.01–0.16]**	**0.036**		
CRP			**0.001 [0.00–0.006]**	**0.035**				
Leucocytes							0.15 [−0.12–0.29]	0.064
R^2^	0.150		**0.523**		**0.050**		0.089	

*CI* confidence interval (μkat/L), *GGT* Gamma-glutamyl transferase (μkat/L), *AP* Alkaline phosphatase (μkat/L), *ALT* Alanine aminotransferase; B: constant in the model R^2^ determination coefficient Creatinine, glucose and Intensive care unit stay do not feature in this model. Bold type indicates significance

Bold means statistically significant

Infection, days of PN, and surgery were related to the elevation of 3 of the LTs studied (GGT, AP and ALT). The variable days of study had noticeable effect in the variation of AP. In fact, the mean number of days it took patients to present AP values above 2-fold their normal value was 13.94 ± 15.03 with statistically significant differences (*p* < 0.05) in patients who did not present this elevation (6.81 ± 4.40 days) (Fig. 1). The mean number of days it took patients to present GGT values above twice their normal value was 7.89 ± 8.03. No statistical differences were found with patients who did not present this elevation (7.03 ± 5.20 days) (Fig. 2). The mean number of days it took patients to present TB values above their initial value was 8.92 ± 10.04 with a tendency to significance (*p* < 0.11) compared with those who did not present this elevation (6.88 ± 3.75 days). Finally, high values of CRP were only significantly associated with increases in AP, while high values of leukocytes only had a tendency to be related to TB elevation (Table 6).

## Discussion

Our results indicate that clinical situations associated with oxidative stress, inflammation and days of PN treatment are independent risk factors in the early onset of impaired hepatic function. As far as we know, this is the first time that this complication has been studied in a group of non-critical patients, homogeneous with regard to PN (same lipid composition same LE adjusted dose per kg, normal initial LT values), and without oral or enteral nutrition intake. The selection criteria that we used in the study attempted to pre-determine the risk factors more readily associated with the early alteration of LTs in hospitalized adult patients, by controlling nutritional risk factors. In this context, it is relevant to study comorbidities in stressed patients, as these involve catabolic and inflammatory states that require supportive and therapeutic interventions associated with LT alterations, making it difficult to disaggregate the cause. In addition to these interventions, the comorbidities themselves, with which these patients usually present, may also be associated with LT alterations [12], although their impact is not so clearly established.

With the aim of minimizing the effect of lipids on liver function, we studied patients with baseline normal LTs who received the same lipid content and proportions (olive/soy) at the same dose (0.8 g/kg/day). However, since we could not rule out the effect of lipid accumulation, which is different for each patient, we introduced the variable days of study. Despite the short duration, in the multivariate model, the length of treatment with PN increased significantly with GGT and AP increases.

At the end of the study, like other authors describe [1, 4, 5], GGT and AP doubled their normal value in 47 and 10.5% of the patients, respectively. The short period studied (8.43 ± 6.64 days) would explain the low incidence in the alteration of ALT and TB, as per our previous studies [10, 13] and other reference publications [14–17]. Such increases of GGT and AP emerge as early indicators of hepatic dysfunction [15], whilst ALT and TB are linked to hepatocellular damage and intrahepatic cholestasis [15–17].

Critical patients and those on prolonged PN treatment were not included in our study. However, an association was found between infection and significantly elevated plasma levels of AP and ALT. In a large series of critical patients, these findings support a relationship between sepsis and LT alteration [18]. Also in a series of newborns treated with PN after abdominal surgery, episodes of sepsis were associated with a 30% increase in bilirubin levels [19] indicative of the bacterial and fungal infections’ associated with cholestasis. And it has been reported that infections are associated with the development of liver fibrosis in children who receive early PN irrespective of the duration of PN, mode of administration, and underlying disease [1, 20, 21]. Possible mechanisms are the capacity of bacteria to transform chenodeoxycholic acid in to lithocolic acid, a more hydrophobic and hepatotoxic biliary acid [22]; accumulation of toxins in the liver due to sepsis condition leading to activation of the Kupffer cells and ultimately to hepatic injury [23] or an inflammatory response in the liver by endotoxin-activated proinflammatory cytokine release that may alter the function of the biliary canaliculum membrane and reduce biliary flow.

Overexpression in the liver of proteins related to oxidative regulation and cellular protection against oxidative stress (peroxiredosin-4, serotransferrin, glutathione S-transferase P and Mn-Superoxide dismutase) suggests that oxidative damage may be one of the main mechanisms to explain liver tissue damage [24]. In animal models, Fas-mediated mitochondrial changes and apoptosis also point to hepatic injury due to oxidative stress [25]. In our population, a high percentage of patients had had surgery (79.6%) and, therefore certain levels of oxidative stress associated with the surgery itself as well as with anesthesia [26].

More than half the patients studied (66.9%) had a diagnosis of cancer. Free radicals are not only implicated in tumor development [27–29], the redox status of cells may influence the activation of genes [28, 29]. High levels of reactive oxygen species, which are associated with oxidative stress and inflammatory processes, are seen in hepatic disease [30]. In fact, in the four multivariate models studied, cancer was included for GGT and AP models, and was statistically significant in the GGT model. These results support the hypothesis of the influence of neoplastic oxidative stress on LD together with the effects of therapies and comorbidities associated with cancer.

In this study, we included the inflammatory parameters CRP and leukocytes, since high levels of reactive oxygen species, which are associated with oxidative stress and inflammatory processes, are seen in hepatic disease [27]. During acute inflammation, a series of chemical and cellular reactions trigger potent vascular changes (vasodilatation, increased permeability), resulting in increased exudate. These events are regulated exclusively by mediators of inflammation related to increases in CRP, among others. Simultaneously, leukocyte activation, induced by chemotactic factors, occurs by phagocytosis and/or antigen-antibody complexes. The final step in phagocytosis occurs mainly through oxygen-dependent mechanisms that generate ROS.

The first limitation of this study is that, given the multifactorial component of the alterations studied, it is difficult to differentiate between the possible causes. However, the restrictive inclusion criteria make possible a better disaggregation of early indicators of LT alterations in adult patients. Another study limitation is that changes in parameters are studied but not their impact on liver function because in clinical practice no invasive techniques could be contemplated in this population. A third methodological limitation is the fact that we have taken the surrogate variable days of PN as an indirect indicator of the lipid accumulation effect.

Since the clinical conditions and characteristics of the PN process (lipid overload and absence of oral/enteral intake) determine the early alteration of LTs, it is important to design therapeutic approaches that minimize long-term risk.

The results obtained here would help to reveal preventive strategies in the initial stages of PN treatment, such as the use of LE derived from other types of LE such as fish oil with an anti-inflammatory, antioxidant and non-phytosterol component.

Finally, we have seen that LF variables are affected by surgical, neoplastic and infectious processes. Recently it has been shown that the administration of phytosterols, specifically stigmasterol, could act by promoting liver inflammation by activating hepatic macrophages and acting as TLR agonists (Toll-like receptors) that induce cytokines [31] concluding that phytosterols by themselves are not associated with liver inflammation but act in the presence of sepsis [32]; this synergy would be one of our research direction.

## Conclusions

In hospitalized adult patients treated with short-term PN, with normal initial LTs and receiving the same lipid composition at the same dose, there is an elevation of LTs, which is more marked for GGT and AP. The remaining biochemical and hematological parameters do not alter or evolve towards normality.

The risk factors that promote this early elevation of some LTs are events such as surgery-, cancer- and infection-associated oxidative stress, as well as inflammatory response (CRP and leukocytes) and days on PN treatment. Thus, surgery is associated with elevation of 3 of the LTs. The duration of PN could probably be linked to the cumulative effect of the administered lipids.

## Abbreviations

ALT: Alanine aminotransferase
ANOVA: Analysis of variance
AP: Alkaline phosphatase
BMI: Body max index
CRP: C-Reactive Protein
GGT: Gamma-glutamyltransferase
HPN: Home parenteral nutrition
LD: Liver disease
LE: Lipid emulsion
LT: Liver test
PN: Parenteral nutrition
PNALD: Parenteral nutrition associated liver disease
TB: Total bilirubin
